# Process Optimization for the Preparation of the Lithium Iron Phosphate Precursor FePO_4_·2H_2_O by Anodic Oxidation Method

**DOI:** 10.3390/ma18112555

**Published:** 2025-05-29

**Authors:** Yang Shao, Ziyuan Liu, Chengping Li, Ying Liu, Zhengfu Zhang, Rundong Wan, Jinsong Wang, Xiaoping Yang, Rui Bao, Yingjie Zhang, Jianhong Yi, Peng Dong, Ding Wang

**Affiliations:** 1 Faculty of Materials Science and Engineering, Kunming University of Science and Technology, Kunming 650093, China; 2 Faculty of Metallurgical and Energy Engineering, Kunming University of Science and Technology, Kunming 650093, China

**Keywords:** anodic oxidation, uniform experimental design, morphology control, particle size optimization, lithium iron phosphate

## Abstract

Iron phosphate (FePO_4_·2H_2_O) was synthesized via anodic oxidation using nickel–iron alloy composition simulates from laterite nickel ore as the anode and graphite electrodes as the cathode, with phosphoric acid serving as the electrolyte. A uniform experimental design was employed to systematically optimize the synthesis parameters including voltage, electrolyte concentration, electrolysis time, and degree of acidity or alkalinity (pH). The results indicate that the addition of cetyltrimethylammonium bromide (CTAB) surfactant effectively modulated the morphology of the anodic oxidation products. The optimized conditions were determined to be an electrolyte concentration of 1.2 mol/L, a voltage of 16 V, a pH of 1.6, an electrolysis time of 8 h, and a 3% CTAB addition. Under these conditions, the synthesized FePO_4_·2H_2_O exhibited enhanced performance as a lithium-ion battery precursor. Specifically, the corresponding LiFePO_4_/C cathode delivered an initial discharge capacity of 157 mA h g^−1^ at 0.2 C, retaining 99.36% capacity after 100 cycles. These findings provide valuable insights and theoretical foundations for the efficient preparation of iron phosphate precursors, highlighting the significant impact of optimized synthesis conditions on the electrochemical performance of lithium iron phosphate.

## 1. Introduction

The escalating global greenhouse gas emissions have intensified climate change and environmental challenges, necessitating the development of green, environmentally friendly, and safe energy sources [1,2,3]. Lithium-ion batteries (LIBs), particularly lithium iron phosphate (LiFePO_4_, LFP) cathodes, have gained prominence due to their abundant raw materials, safety, stability, low cost, low toxicity, and long cycle life, making them ideal for new energy vehicles and other applications [4,5,6,7]. Various synthesis methods for LFP have been developed, including carbothermal reduction [8], high-temperature solid-phase synthesis [9], microwave synthesis [10], co-precipitation [11], hydrothermal synthesis [12], solvothermal synthesis [13], the sol–gel method [14], and spray drying techniques [15], among these, carbothermal reduction is widely regarded as economical and convenient, utilizing inexpensive precursors and forming uniform carbon coatings that enhance performance [6,16,17,18,19].

The performance of iron phosphate precursors significantly affects the electrochemical properties of the resulting LFP cathodes. Previous studies have reported various methods to synthesize FePO_4_ with controlled morphology and particle size, which in turn influence the electrochemical performance of LFP [20]. Fine FePO_4_ with a uniform particle size was prepared using a hydrothermal approach; the prepared FePO_4_ was subjected to encapsulation and modification with three-dimensional porous graphene (3DG) to produce a precursor. V^2+^ or V^4+^ ions were incorporated during lithiation, and a similar material was subsequently subjected to calcination to obtain the final cathode material LiFe_0.98_V_0.02_PO_4/3_DG/C, which exhibited exceptional electrochemical properties. Furthermore, the influence of precursor preparation under varying pH conditions on the performance of LiFePO_4_/C cathode materials was investigated. Lin et al. [21] utilized iron scraps as raw materials and employed electrolytic anodic flocculation to synthesize FePO_4_ with various particle sizes and morphologies by adjusting pH. The resulting FePO_4_ was used as a precursor to prepare a LiFePO_4_/C material exhibiting excellent electrochemical performance. Ma et al. [22] recovered iron phosphate from the phosphorus-rich waste of fish bone ash extract via electrodeposition; moreover, they employed the response surface methodology with the Box–Behnken design to optimize the acid leaching conditions (molar concentration, time, and ash amount). Kaikake et al. [23] adopted an electrochemical approach to synthesize amorphous mesoporous iron phosphate with a high specific surface area. The LiFePO_4_/C produced using this material as a precursor exhibited exceptional electrochemical properties. This methodology offers a novel approach for the synthesis of other mesoporous phosphates. Yu, et al. [24] synthesized high-quality FePO_4_·2H_2_O at low temperatures by electrodepositing ferrous sulfate in a phosphoric acid solution with an oxidation potential of E > 0.3 V/Saturated Calomel Electrode (SCE). They revealed that the electrodeposition of ferrous sulfate was controlled by diffusion and that a reversible reaction occurred at the electrode interface. On the basis of a thermodynamic analysis and experimental results, Bouamer et al. [25] reported that an iron phosphate precursor with a perfect crystal form, few impurities, and an appropriate iron-to-phosphorus ratio (Fe/P) can be prepared under pH 2.4 conditions and with the addition of an oxidant. Jugović et al. [26] systematically examined the effects of surfactant type, dosage, aging time, and ultrasonic time on the preparation of iron phosphate via rapid precipitation and determined the optimal conditions for this preparation process. These conditions were as follows: the addition of the surfactant cetyltrimethylammonium bromide (CTAB) in an amount equivalent to 1.5% by mass of iron powder, aging for 4 h, and ultrasonic treatment for 60 min. Thus, rapid precipitation is a feasible method for preparing industrial-grade iron phosphate.

In this study, we employed a combination of a uniform experimental design and single factor experiments to systematically investigate the factors influencing the anodic oxidation synthesis of FePO_4_·2H_2_O. By optimizing parameters such as voltage, electrolyte concentration, pH, and electrolysis time, and by adding a CTAB surfactant, we aimed to control the morphology and particle size of FePO_4_·2H_2_O. The optimized FePO_4_·2H_2_O was then used as a precursor to synthesize LFP/C cathodes, and the effects of precursor properties on the electrochemical performance of LFP were evaluated. This study provides important experimental evidence and theoretical support for the efficient synthesis of iron phosphate precursors and the development of high-performance LFP cathode materials.

## 2. Materials and Methods

The materials used in this study were obtained as follows: anode material: nickel–iron alloy composition simulates from laterite nickel ore (Yunnan Province, China); electrolyte: diluted phosphoric acid (H_3_PO_4_, 85%); cathode material: graphite electrode; surfactant: cetyltrimethylammonium bromide (CTAB, AR); and other chemicals: lithium propionate (CH_3_CH_2_COOLi; AR), hydrogen peroxide (H_2_O_2_, 30wt.%), ascorbic acid (C_6_H_8_O_6_, AR), N-methyl-2-pyrrolidone (NMP, AR), polyvinylidene fluoride (PVDF, 99.99%), and conductive carbon black (industrial grade). The experimental flowchart is shown in Figure S11.

This experiment employs a nickel–iron alloy that is analogous to the composition of laterite nickel ore found in the Yunnan Province. The sample is first abraded using sandpaper, followed by a thorough cleansing with anhydrous ethanol. Subsequently, the sample undergoes a drying process in a blast drying oven set at a temperature of 60 °C for a duration of 12 h. The Fe_0.64_Ni_0.36_ alloy composition was confirmed by XRF (Table 1). The absence of distinct XRD peaks (Figure S10) stems from the nanocrystalline surface layers (<50 nm grain size, Figure S2) formed during mechanical abrasion, as described by the Scherrer equation. Impurities (<1%) were verified via Inductively Coupled Plasma Optical Emission Spectrometry (ICP–OES) (Table S1) and do not contribute significantly to the diffraction pattern. This raw material was identified as an Fe_0.64_Ni_0.36_ alloy due to the presence of other impurity elements, which were found to be less than 1%. Consequently, the diffraction peaks could not be observed in the XRD pattern.

FePO_4_·2H_2_O was synthesized via a simple two electrode anodic oxidation process using a DC power supply (MS303D, Maisheng Power Technology, Dongguan, Guangdong, China). The Ni–Fe alloy anode (80 × 20 mm) and graphite cathode (80 × 20 mm) were immersed in a diluted phosphoric acid electrolyte. The 85% H_3_PO_4_ (14.7 mol/L, Sigma-Aldrich, St. Louis, MO, USA) was diluted with deionized water to achieve the target concentrations (0.1–2 mol/L). The pH was subsequently adjusted to the desired value (0.8–3.2) by a dropwise addition of NH_3_·H_2_O and H_3_PO_4_ under continuous stirring, ensuring the final H_3_PO_4_ concentration remained within ±2% of the design value. The electrolyte temperature was maintained at 60 °C using a water bath system to ensure consistent reaction conditions. A uniform experimental design was implemented using a DPS data processing system to optimize synthesis parameters. Factors such as electrolyte concentration (0.1–2 mol/L), voltage (8–16 V), pH (0.8–3.2), and electrolysis time (1–9 h) were systematically varied (Table 2). After anodic oxidation, 30% H_2_O_2_ was added to oxidize Fe^2+^ to Fe^3+^, promoting the formation of an FePO_4_·2H_2_O precipitate. The anodic oxidation product FePO_4_·xH_2_O (x ≈ 1.8–2.2) is converted to phase-pure FePO_4_·2H_2_O through controlled drying (80 °C, 12 h), as validated by TG mass loss (18.63%) and XRD (PDF #33-0667). The stoichiometric 2H_2_O designation reflects crystallographic ally bound water.

The experimental conditions set for this experiment were as follows: electrolyte concentration: 0.1–2 mol/L; voltage: 8–16 V; pH: 0.8–3.2; and duration of electricity supply: 1–9 h. A homogeneous experimental design table was designed from the described experimental factors and variable levels as shown in Table 2. After the completion of the reactions in each group, a 30% H_2_O_2_ solution was slowly added to the suspension and the suspension was stirred for 1 h. Subsequently, the precipitate was filtered and washed multiple times and then placed in a blast-drying oven for drying at 80 °C for 12 h. Finally, the FePO_4_·2H_2_O sample was obtained.

The obtained FePO_4_·2H_2_O samples were mixed with lithium acetate (Fe/Li molar ratio of 1:1.05) and 10 wt.% ascorbic acid as a carbon source. The mixture was dispersed in 70 mL anhydrous ethanol and stirred at 70 °C for 6 h. After drying, the mixture was sintered at 650 °C for 8 h under an argon atmosphere to obtain LiFePO_4_/C samples.

The crystallinity of the sample was examined by X-ray diffraction (XRD diffractometer Ultima IV Rigo, Rigaku Corporation, Tokyo, Japan). Test conditions were as follows: target material: Cu target; tube voltage: 35 kV; tube current: 30 mA; and scanning speed: 5°/min. The surface of FP material was analyzed using a scanning electron microscope (SEM, S-4800, Hitachi, Tokyo, Japan). The surface morphology of the FP material was analyzed and observed by transmission electron microscopy (TEM FEI Talos F200X, Thermo Fisher Scientific, Waltham, MA, USA), and the content of each element in the FP material was quantitatively analyzed by an inductively coupled plasma optical emission spectrometer (ICP-OES AGILENT 725-ES, Agilent Technologies, Wilmington, DE, USA). The content of the crystalline water in the FP material was determined by a thermogravimetric thermal difference analysis (TG-DSC STA6000, PerkinElmer, Waltham, MA, USA) at a heating rate of 10 °C/min. The test temperature range was 30–800 °C.

To further evaluate the performance of the iron phosphate precursor, a LiFePO_4_/C cathode material was prepared from the precursor and assembled into 2025-coin cells. First, LiFePO_4_/C, acetylene black, and PVDF were mixed in at a mass ratio of 8:1:1 with N-methyl-2-pyrrolidone to form a black slurry, which was then coated on aluminum foil. Subsequently, cathode material pellets (diameter: 12 mm) were prepared. Next, the cathode pellet, lithium metal foil, 1M LiPF_6_/EC-EMC-DMC electrolyte (with a volume ratio of 1:1:1), and Polyethylene battery separator were assembled into the battery in an argon-filled glovebox. The first charge/discharge performance and 100-cycle performance were tested using a battery testing system (CT-3008W-5V-10mA, Neware, Shenzhen, China; voltage window: 2.5–4.2 V, and 1C rate: 170 mA h g^−1^). Cyclic voltammetry (CV) tests were performed at a scan rate of 0.5 mV s^−1^. Electrochemical impedance spectroscopy (EIS) tests were conducted on both pre-cycling and post-cycling LFP cells.

The battery assembly process is as follows: first, the negative shell containing the centrally positioned lithium plate is placed in the glove box. Next, 55 μL of electrolyte is added, followed by placing the separator on top. The lithium plate is subsequently positioned over the separator, and the positive electrode is placed thereafter. A second addition of an electrolyte is performed, after which the gaskets and shrapnel are added sequentially. Finally, the positive shell is placed, and the battery is sealed using an encapsulation machine (Crystal Precision Manufacturing, Shanghai, China). The assembled battery remains in the glove box for 24 h before being removed for the testing of its charging and discharging performance.

## 3. Results and Discussion

### 3.1. Synthesis Mechanism of FePO_4_·2H_2_O

**Figure 1:**
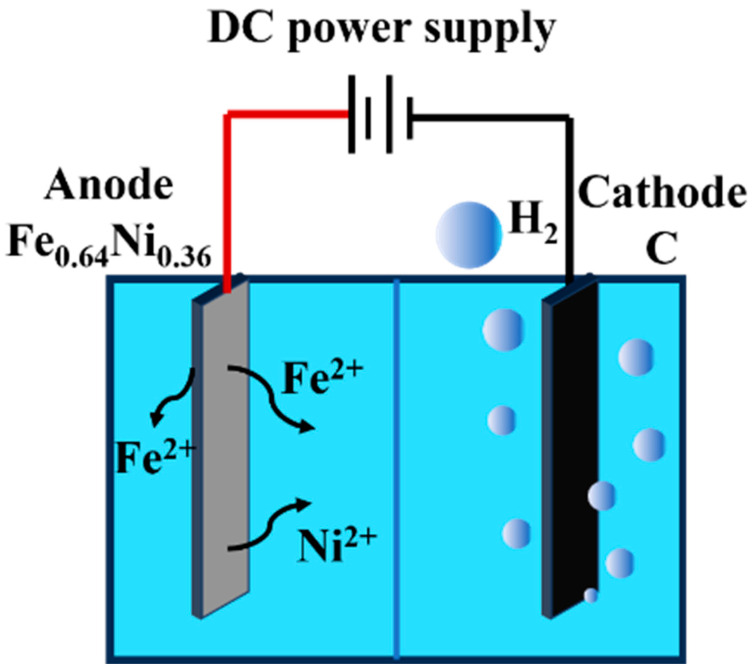
Mechanism of anodic oxidation.

Fe and Ni are located in the eighth group of the fourth period in the periodic table. Both elements have two outer electrons in their outermost orbitals, making it easy for them to lose these electrons to form Fe^2+^ and Ni^2+^ during electrochemical reactions [12]. After completion of anodic oxidation, 30% hydrogen peroxide is added to oxidize Fe^2+^ to Fe^3+^. The completeness of Fe^2+^ oxidation by H_2_O_2_ is thermodynamically ensured by the redox potential difference. The standard oxidation potential of H_2_O_2_ (E^0^ = +1.76 V vs. SHE) significantly exceeds that of Fe^2+^/Fe^3+^ (E^0^ = +0.77 V vs. SHE). With 30% H_2_O_2_ in 5-fold stoichiometric excess and a 1 h reaction time, the Gibbs free energy change (ΔG = −237 kJ/mol) confirms spontaneous and complete oxidation, consistent with prior reports [25]. Ni already has eight electrons in the next-to-outermost orbital; hence, no change occurs in the oxidation state of Ni^2+^. In the solution, Fe^3+^ combines with PO_4_^3−^ to form an FePO_4_·xH_2_O precipitate; moreover, Ni^2+^ remains in the ionic form under acidic conditions and does not form a precipitate with PO_4_^3−^ [17]. This is because the solubility of iron phosphate is considerably low in acidic environments. When PO_4_^3−^ binds to Fe^3+^, the resulting iron phosphate precipitate has low solubility, leading to precipitation in the solution. In comparison, nickel phosphate has higher solubility and remains stable in the solution without forming a precipitate. The mechanism of the electrochemical anodic oxidation reaction is shown in Figure 1.

During anodic oxidation, Fe atoms in the Ni–Fe alloy anode lose electrons to form Fe^2+^ ions, while Ni remains as Ni^2+^ due to its stable electronic configuration. The reactions are as follows:

Anode: Ni_0.36_Fe_0.64_ → 0.64Fe^2+^ + 0.36Ni^2+^ + 2e^−^ (1)

Cathode: 2H^+^ + 2e^−^ → H_2_↑ (2)

Overall reaction equation: Ni_0.36_Fe_0.64_ + 2H^+^ → 0.64Fe^2+^ + 0.36Ni^2+^ + H_2_↑ (3)

Based on the experimental performance and subsequent Inductively Coupled Plasma (ICP) tests, Ni^2+^ is retained in the experimental waste solution.

After electrolysis, H_2_O_2_ oxidizes Fe^2+^ to Fe^3+^, which then reacts with PO_4_^3−^ ions to form an FePO_4_·2H_2_O precipitate:

Precipitation reaction equation: Fe^2+^ + PO_4_^3−^ + xH_2_O → FePO_4_·xH_2_O↓ (4)

Ni^2+^ remains in the solution due to the higher solubility of nickel phosphate under acidic conditions.

### 3.2. Single Factor Experiments

Temperature plays a crucial role in controlling the morphology and particle size of FePO_4_·2H_2_O. Excessive temperature can lead to increased ion collision rates, causing uneven particle size distribution and agglomeration. By controlling the electrolyte temperature at 60 °C, we achieved optimal conditions for the formation of FePO_4_·2H_2_O with the desired morphological characteristics. A circulating water bath system was used to control the electrolyte temperature cell while keeping other conditions constant (electrolyte concentration: 1 mol/L and constant voltage: 16 V). The selected temperatures were 40 °C, 50 °C, 60 °C, and 70 °C, and the corresponding iron phosphate samples were designated as FP-40, FP-50, FP-60, and FP-70, respectively.

**Figure 2:**
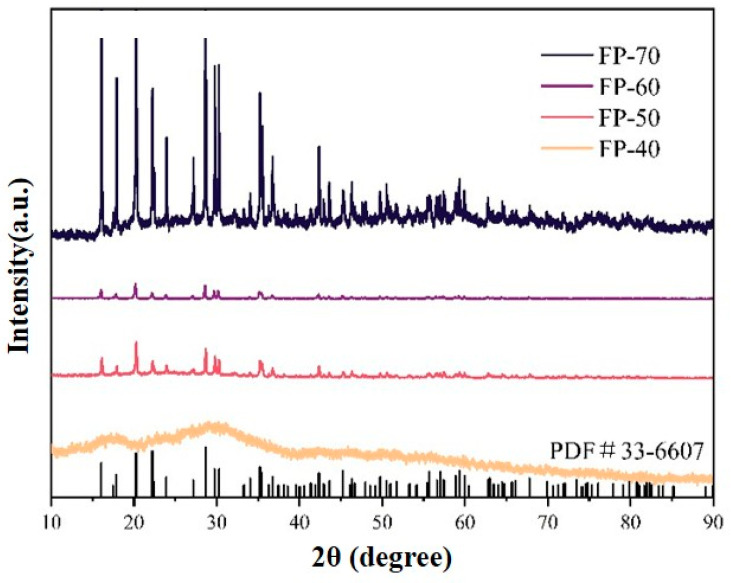
XRD patterns of anodic oxidation products under different water bath temperature conditions.

As shown in Figure 2, the electrolyte temperature considerably affects the crystallinity of the anodic oxidation precursors. At 40 °C, the precursor is amorphous, and at 50 °C, it is both crystalline and amorphous. Moreover, at 60 °C, it is completely crystalline. At 70 °C, the diffraction peaks become more intense and broader than those at 60 °C, indicating the formation of numerous small crystallites. Lower temperatures provide insufficient energy for nucleation, resulting in the formation of amorphous iron phosphate. The crystallinity improves with the increasing temperature. When the temperature reaches 70 °C, many nucleation sites are created because of the increased energy, leading to smaller crystal sizes owing to the rapid anodic oxidation reaction.

**Figure 3:**
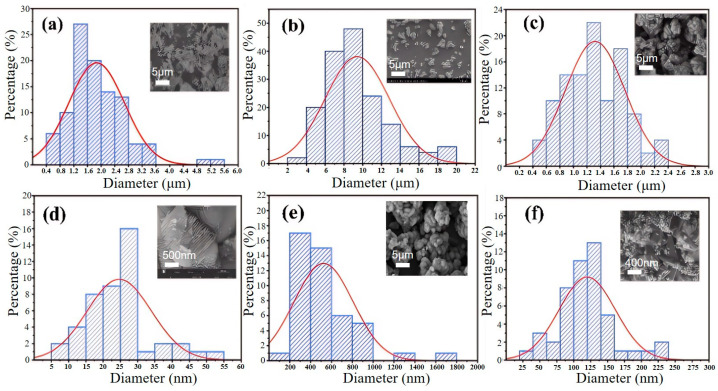
Particle size distribution chart of anodic oxidation products under different water bath temperature conditions: (**a**) 40 °C, (**b**) 50 °C, and (**c**) 60 °C. (**d**) Distribution of interlayer thickness at 60 °C, (**e**) primary particle size distribution at 70 °C, and (**f**) secondary particle size distribution at 70 °C.

**Figure 4:**
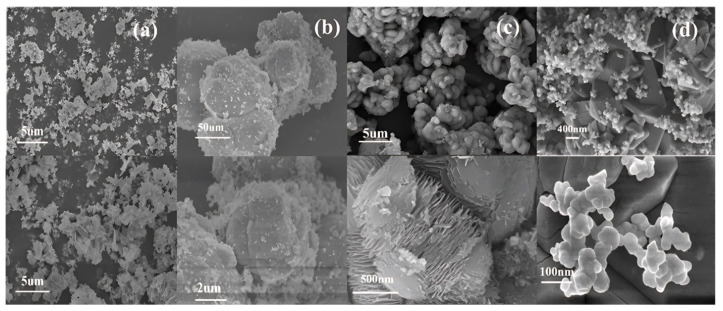
Scanning electron microscopy (SEM) images of anodic oxidation products under different water bath temperature conditions: (**a**) 40 °C, (**b**) 50 °C, (**c**) 60 °C, and (**d**) 70 °C.

Figure 3 and Figure 4 show that the morphology and particle size of iron phosphate vary considerably with the changing water bath temperature. At 40 °C, iron phosphate appears as fragmented irregular flakes with severe agglomeration and an average particle size of 1.8 μm. At 50 °C, particle morphology tends to be relatively spherical and the average particle size increases to 9 μm; however, the larger particle size may adversely affect the electrochemical performance of the synthesized lithium iron phosphate. At 60 °C, the average particle size decreases to 1.3 μm, with a layer thickness of 26 nm, demonstrating its suitability as a precursor for lithium iron phosphate. At 70 °C, the primary particles (average size: 450 nm) and secondary particles (average size: 135 nm) were observed; this uneven particle size distribution may create inhomogeneity in the lithium iron phosphate composition, adversely affecting the performance consistency of different batches of products.

**Figure 5:**
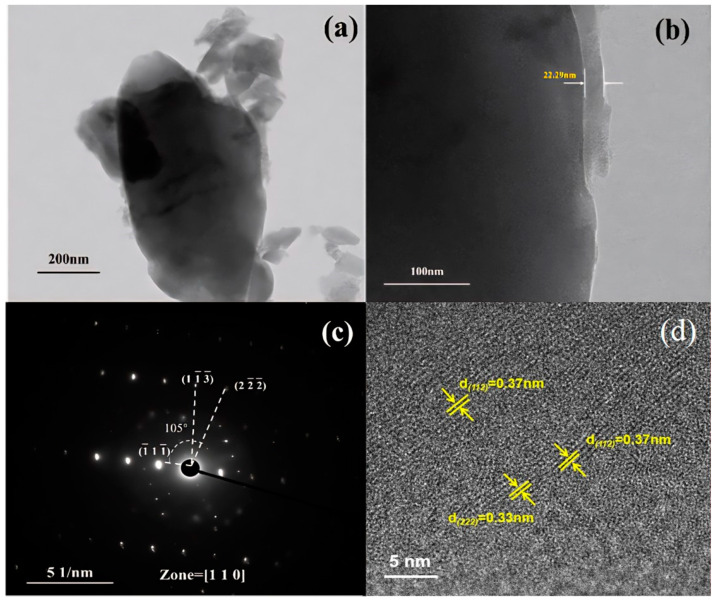
(**a**,**b**,**d**) Transmission electron microscopy (TEM) images of FP-60 at different scales and (**c**) electron diffraction pattern of FP-60.

Figure 5 shows that after ultrasonic dispersion, FP-60 comprises nanosheets with an average length and width of approximately 1104 and 456 nm, respectively. The average layer thickness is approximately 26 nm. The FP-60 sample contains crystalline and amorphous regions, and the amorphous region at the edge is approximately 22 nm thick. The crystalline region includes (142), (331), (112), and (221) planes with interplanar spacings of 0.37, and 0.33 nm, respectively. The electron diffraction pattern presented in Figure 5 reveals planes ($\bar{1}$1$\bar{1}$), ($\bar{2}$2$\bar{2}$), and (1$\bar{1}$$\bar{3}$), establishing the crystal zone axis as [110]. The FP-60 sample has a layered morphology; therefore, its grains do not considerably grow in the [110] direction.

### 3.3. Optimization via Uniform Experimental Design

The nine sets of experimental protocols obtained from the experimental design table presented in Table 2 are named Scheme: FP 1-FP 9 (Table 3). Using DPS v21.05 software [27], we designed a uniform experimental matrix to explore the interactive effects of synthesis parameters. The optimized conditions were found to be the following: electrolyte concentration: 1.2 mol/L, voltage: 16 V, pH: 1.6, and electrolysis time: 8 h. Under these conditions, FePO_4_·2H_2_O exhibited improved crystallinity, a uniform morphology, and controlled particle size distribution.

Different types of surfactants were added to the electrolyte for anodic oxidation at 1% of the mass of the iron source to prepare iron phosphate by an anodic oxidation reaction. The surfactants selected for the experiment were the following: the cationic surfactant CTAB, the anionic surfactant SDBS, and the polymeric surfactant PVP. The structural formula of each of the above surfactants is shown in Figure S4.

As demonstrated in Figure S5, a comparison was conducted between the XRD patterns of the anodic oxidation products obtained following the addition of various surfactants and the standard PDF card (PDF #33-0667). The analysis revealed that the predominant components of the products were identified as FePO_4_-2H_2_O. However, a notable observation was the presence of Fe(H_2_PO_4_)_3_-2H_2_O. The presence of this phase can be attributed to the ability of the pyrrolidone unit in the PVP molecule to form a ligand bond with the metal ion through the oxygen atom. This interaction has the potential to alter the reaction kinetics between Fe^2+^ and H_2_PO^4−^ and thereby promote the formation of the Fe(H_2_PO_4_)_3_-2H_2_O phase. Secondly, the dissolution of PVP in water will form a certain viscosity, which may affect the ion migration and diffusion process in the electrolyte. Under the action of PVP, this results in the emergence of a local concentration gradient in the electrolyte, thus affecting the direction of the reaction and the formation of the product. The anodic oxidation of the product obtained by the addition of CTAB and SDBS has no obvious heterogeneous peaks, and the composition is relatively homogeneous. In the sample with CTAB, the diffraction peaks were found to be sharper and stronger, indicating that the crystallinity of the product was higher. In the sample with SDBS, compared with the CTAB sample, the half-height width of the diffraction peak was found to be relatively narrow, and the half-height width was found to be inversely proportional to the grain size, indicating that the grain size of the SDBS sample was relatively large compared with that of the CTAB sample under the same additive ratio conditions.

As demonstrated in Figures S6 and S7, the morphology of the resulting anodic oxidation products of iron phosphate varies significantly under different surfactant addition conditions. When SDBS is added to the electrolyte, the surface morphology of the product is a regular prismatic block piled up by thicker lamellae, and the lamellae are more uniformly distributed. The average particle size of SDBS-FePO_4_ is 3 μm, and the average thickness of the lamellae is 125 nm. The morphology of the anodic oxidation product obtained from the addition of PVP to the electrolyte is an irregular block with lamellar particles, and the product obtained from the addition of the surfactant is more prone to agglomeration, with an average particle size of 0.9 μm. The average particle size of the CTAB-FePO_4_ was found to be 100 nm, with an average flake thickness of 30 nm. The sample obtained by using this surfactant reached the nanometer scale compared with the former two, and it was the smallest size of the particles in the three samples. However, the sample exhibited a problem of serious agglomeration. In this experiment, ICP-OES was used to analyze the content of each element, the iron/phosphorus ratio and the content of the impurity Ni^2+^ in the samples obtained from different CTAB additions, as shown in Table S3.

**Figure 6:**
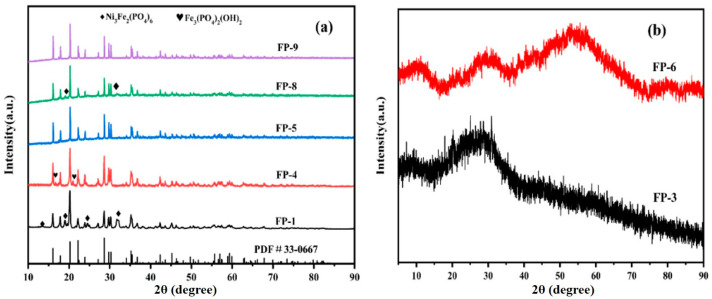
XRD patterns of the products obtained using a uniform experimental: (**a**) crystalline anodized oxide and (**b**) amorphous anodized oxide.

The crystallinity, crystal structure, and space group of the anodic oxidation products obtained using a uniform experimental design were analyzed via their XRD patterns. The XRD patterns of the samples are shown in Figure 6a,b.

According to Table 3, the homogenization experiment did not yield any anodic oxidation product after the completion of the experiments for schemes FP-2 and FP-7. Precipitate formation is closely related to the solution pH and the solubility product constant. In scheme FP-2, the pH was 0.8; under these highly acidic conditions, the high concentration of H^+^ reacted with PO_4_^3−^ to form highly soluble HPO_4_^2−^ or H_2_PO_4_^−^, thereby reducing the number of PO_4_^3−^ available to react with Fe^2+^ to form FePO_4_ [17]. Hence, no precipitation was observed in this scheme. In scheme FP-7, the solubility product constant was a constant representing the product of the ion concentrations in a saturated solution of solid salt under specific temperature conditions.

In scheme FP-7, owing to the short duration of electrolysis, the concentration of Fe^2+^ in the electrolyte was low and the ion concentrations in the solution did not exceed the solubility product constant. Hence, no precipitation was observed. The XRD patterns of samples FP-3 and FP-6 obtained from schemes FP-3 and 6 indicated an amorphous state. The amorphous nature of sample FP-3 may be attributed to the fact that the initial iron phosphate product prepared via anodic oxidation was amorphous. Owing to the short electrolysis reaction time, the product did not have sufficient time for transformation into a stable crystalline form.

Sample FP-6 was prepared in a 0.1-mol/L dilute phosphoric acid solution with a pH of 1.3. Owing to the low electrolyte concentration, the dilute phosphoric acid solution provided a limited number of PO_4_^3−^. When Fe^2+^ entered the electrolyte, the insufficiency of PO_4_^3−^ prevented complete binding with Fe^2+^. Hence, precipitate formation occurred in the amorphous state. Additionally, the low pH of the electrolyte in the anodic oxidation reaction led to competition between H^+^ and Fe^2+^ for reaction sites with PO_4_^3−^, reducing the number of PO_4_^3−^ available to form ordered crystals with Fe^2+^. Hence, the final reaction product was amorphous [10].

Figure 5, Figures S2 and S3 show that the morphology of sample FP-1 obtained via scheme FP-1 is characterized by irregular flakes with an average width of 0.45 μm. However, this sample exhibits considerable agglomeration of the flakes. Samples FP-3 and FP-6, as shown in Figure 6b, are amorphous. The products obtained in schemes FP-3 and 6 exhibit severe agglomeration and have irregular block-like morphologies, with average particle sizes of 5 and 1.8 μm, respectively. Sample FP-4 obtained via scheme FP-4 has a morphology comprising unevenly sized spherical particles with an average grain size of 1.1 μm. Sample FP-8 obtained via scheme FP-8 has a blocky morphology characterized by the presence of secondary grains; the average sizes of the primary and secondary grains are 2 and 110 nm, respectively. Sample FP-5 obtained via scheme FP-5 has a flaky morphology, with an average flake size of 120 nm; however, this sample also exhibits considerable agglomeration, with agglomerate sizes reaching 8 μm. Sample FP-9 obtained via scheme FP-9 has a spherical morphology comprising flakes, with an average spherical particle size of 4 μm and an average flake thickness of 200 nm.

The elemental compositions and impurity levels, specifically the Ni^2+^ content, of samples FP-1–FP-9 obtained via the homogenization experiments were analyzed using Inductively Coupled Plasma Optical Emission Spectrometry (ICP–OES), and the Fe/P was calculated for each sample (Tables S1 and S2). The elemental concentrations and Fe/P of the samples are presented in Supporting Information Table S1. Fe/P was calculated as shown in Equations (5) and (6). Figure 6 shows the elemental composition of the products of the homogenization experiments. According to the standard HG/T 4710-2021, the Fe/P for battery-grade iron phosphate should be 0.96–1.02. However, the Fe/P ratios of the iron phosphate sample (FP-1, FP-3, FP-4, FP-5, FP-6, and FP-8) fall below the minimum threshold set by the industry standard, indicating that these samples do not meet the Fe/P specifications required for battery-grade iron phosphate. In contrast, sample FP-9 has an Fe/P of 1.006, which meets the industry standards for battery-grade iron phosphate, and its Ni^2+^ content is 0.14%, reflecting a considerably low level of impurities. The elemental compositions of the products of the homogenization experiments are presented in Supporting Information Tables S1 and S2. From Equation (3) and Tables S1 and S2, the results of the experiments make clear that Ni^2+^ is retained in the electrolyte at the end of the reaction.

(5) $$C_{m}=\frac{Cp\times m0}{M_{p}}$$

(6) $$I=\frac{C_{Fe}}{C_{p}}$$

where *C_m_* is the molar concentration of each element (mol/kg), *Cp* is the relative content of the elements tested (%)*, m*_0_ is the total mass of the sample (kg)*, M_p_* is the molar mass of each element, *I* is Fe/P, *C_Fe_* is the molar concentration of iron (mol/kg), and *C_p_* is the molar concentration of phosphorus (mol/kg).

**Figure 7:**
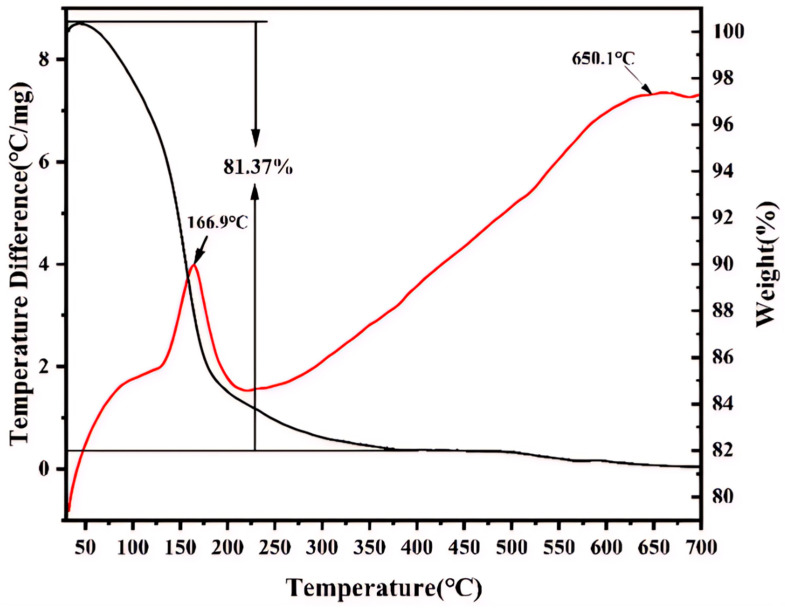
The thermal gravimetric (TG) and differential scanning calorimetry (DSC) curve (TG–DSC curve) of FP-9.

The TG curve shown in Figure 7 indicates that iron phosphate undergoes considerable mass loss in the temperature range of 30–700 °C primarily because of the evaporation of the water of crystallization under high-temperature conditions. The mass loss of iron phosphate is ~18.63%, indicating that 1 mol of FePO_4_ combined with 2 mol of water molecules in this sample. Furthermore, the DSC exotherm at 166.9 °C corresponds to the crystallization of anhydrous FePO_4_ following dehydration, as evidenced by the structural transition in XRD (Figure 6a). While water removal itself is endothermic, the dominant exothermic signal arises from lattice energy release during crystallization, consistent with the reports on hydrated phosphates. At 650.1 °C, an exothermic peak is observed in the DSC curve, while no considerable change is observed in the TG curve, suggesting that a phase transformation occurs at this temperature. Figure 7 shows no mass loss attributable to organic residues beyond 500 °C. These observations confirm CTAB’s role as a transient morphology-directing agent, with no residual surfactant in the final product.

### 3.4. Morphology and Particle Size Control with CTAB

Based on the results of the above experiments, the experimental conditions of scheme FP-9 were used to continue the particle refinement experiments (FP-9 hereinafter is referred to as FP). Adding a CTAB surfactant to the electrolyte significantly influenced the growth of the FePO_4_·2H_2_O crystals. With 3% CTAB, we obtained FePO_4_·2H_2_O with a reduced particle size (~250 nm) and improved uniformity. The surfactant molecules adsorbed on specific crystal facets, hindering unrestricted growth and facilitating controlled morphology. The effects of different types of surfactants on the anodic oxidation products are shown in Figures S4–S8 and Table S3. The effect of CTAB dosage on the iron phosphate prepared via electrochemical anodic oxidation was examined [28]. The single-factor experimental design is presented in Table 4.

Samples with different CTAB dosages were named as follows: FP-0.05CTAB, FP-1.5CTAB, FP-3CTAB, and FP-5CTAB.

**Figure 8:**
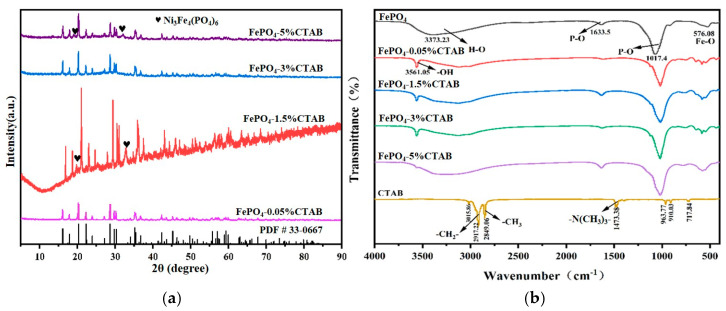
(**a**) XRD patterns of the products of the single-factor experiment. (**b**) Infrared spectroscopy analysis of the samples obtained in the single-factor experiment.

According to Figure 8a, the XRD patterns of the electrolyte with the added CTAB during anodic oxidation show FePO_4_ was the primary component. The XRD patterns of samples FePO_4_-0.05%CTAB, FePO_4_-3%CTAB, and FePO_4_-5%CTAB have sharp diffraction peaks, indicating excellent crystallinity. In comparison, the XRD pattern of FePO_4_-1.5%CTAB indicates lower crystallinity, possibly because of the uneven adsorption of CTAB molecules, the impact of these molecules on electrolyte properties, and changes in the crystal growth rates. Samples FePO_4_-1.5%CTAB and FePO_4_-5%CTAB contain the Ni_3_Fe_4_(PO_4_)_6_ phase, whereas samples FePO_4_-0.05%CTAB and FePO_4_-3%CTAB do not contain any impurities (as indicated by the absence of impurity peaks in their XRD patterns). This result is related to the effect of CTAB concentration on the physicochemical properties of the electrolyte; this effect may alter the local environment during electrolysis and promote the formation of Ni_3_Fe_4_(PO_4_)_6_.

According to Figure 8b, the main peaks in the infrared spectrum of the iron phosphate sample without CTAB are located at 576.08, 1017.4, 1633.5, and 3373.23 cm^−1^, corresponding to the Fe–P stretching vibrations, the P–O stretching vibration, the P–O bending vibration, and the H–O stretching vibration in the crystallized water, respectively. In the infrared spectrum of the samples with CTAB, the peaks observed in the range of 2700–3100 cm^−1^ indicate the stretching vibrations of the –CH_3_ and –CH_2_ groups, while the peaks in the range of 1400–1500 cm^−1^ correspond to the –N(CH_3_)_3_ stretching vibrations. In the infrared spectrum of the samples with CTAB, the stretching vibration peak observed at 3561.05 cm^−1^ corresponds to –OH, suggesting that the trimethylammonium group of CTAB serves as a hydrogen bond acceptor, forming hydrogen bonds with the hydrogen atoms in water molecules. This interaction stabilizes and increases the number of water molecules adsorbed on the FePO_4_ surface. When CTAB is adsorbed on the sample surface, it may form hydrogen bonds with the surface –OH groups, enhancing the vibration of the –OH groups and increasing the intensity of the –OH stretching vibration peak. However, when the CTAB concentration reaches 5%, the peak observed at 3561 cm^−1^ weakens, likely because of the formation of a saturated CTAB adsorption layer on the iron phosphate surface. When this layer is saturated, a further addition of CTAB does not increase the amount of surface-adsorbed water. Instead, the excess CTAB may interact with the adsorbed water molecules through hydrophobic interactions, leading to the desorption of water from the surface.

**Figure 9:**
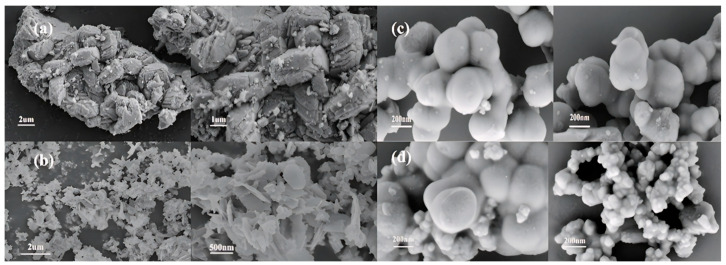
SEM images of iron phosphate obtained with different CTAB dosages: (**a**) FePO_4_-0.05CTAB, (**b**) FePO_4_-1.5CTAB, (**c**) FePO_4_-3CTAB, and (**d**) FePO_4_-5CTAB.

**Figure 10:**
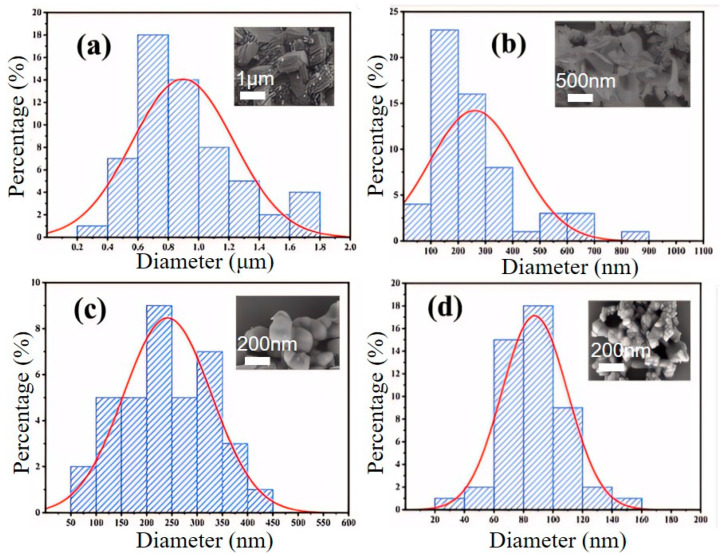
Particle size distribution chart of iron phosphate obtained with different CTAB dosages: (**a**) FePO_4_-0.05CTAB, (**b**) FePO_4_-1.5CTAB, (**c**) FePO_4_-3CTAB, and (**d**) FePO_4_-5CTAB.

As shown in Figure 9 and Figure 10, when the CTAB concentration is 0.05%, the obtained sample FePO_4_-0.05CTAB exhibits a flaky morphology with considerable agglomeration; the largest, smallest, and average flake widths are 1.8, 0.3, and 0.9 μm, respectively. At 1.5% CTAB, sample FePO_4_-1.5CTAB also shows a flaky morphology with low agglomeration; the largest, smallest, and average flake sizes are 850, 80, and 250 nm, respectively. At 3% CTAB, sample FePO_4_-3CTAB comprises spherical particles with maximum, minimum, and average particle sizes of 400, 80, and 250 nm, respectively. At 5% CTAB, sample FePO_4_-5CTAB comprises spherical particles with an agglomeration more severe than in case of the spherical particles in sample FePO_4_-3CTAB; moreover, the largest, smallest, and average particle sizes are 450, 25, and 90 nm, respectively.

A comparative analysis of the elements, iron/phosphorus ratio, and Ni^2+^ impurity in the samples of FP-0.05CTAB~FP-5CTAB obtained from the one-way experiments in Table S3 and Figure S8 was conducted, which shows that the iron/phosphorus ratio of FP obtained when the addition of CTAB is 0.05% is 1.009, which is in line with the iron/phosphorus ratio of battery-grade FP as stipulated in the standard HG/T4710-2021, but the content of impurity Ni^2+^ in the sample is relatively high, at 0.154%. The iron/phosphorus ratios of FP-1.5CTAB and FP-5CTAB are lower than the minimum iron/phosphorus ratio of the battery-grade iron phosphate specified in the standard. The impurity content is relatively high; therefore, they are not suitable for the preparation of lithium iron phosphate cathode materials. The iron-to-phosphorus ratio of FP-3CTAB is 1.001, which is in line with the battery-grade iron phosphate standard, and has a low content of impurity Ni^2+^. This makes it a relatively ideal lithium iron phosphate precursor [29,30,31].

### 3.5. Electrochemical Performance of LiFePO_4_/C Cathodes

The LiFePO_4_/C cathodes synthesized from the optimized FePO_4_·2H_2_O precursors demonstrated an enhanced electrochemical performance.

**Figure 11:**
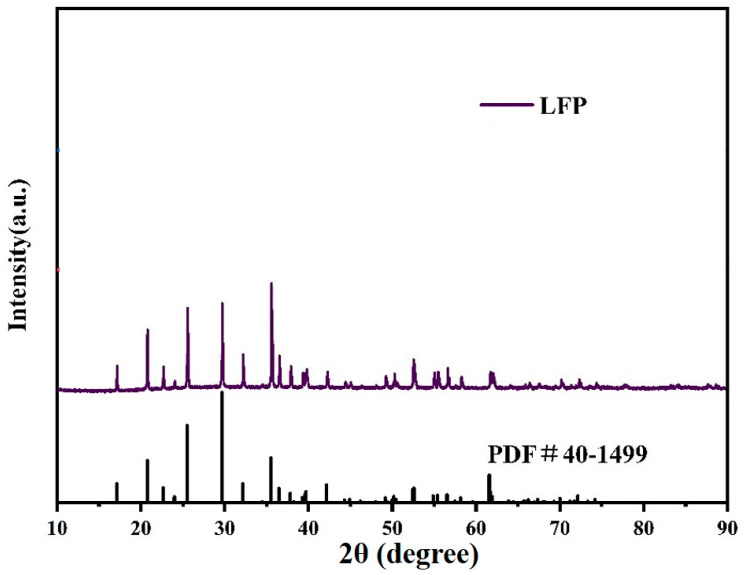
XRD patterns of lithium iron phosphate.

**Figure 12:**
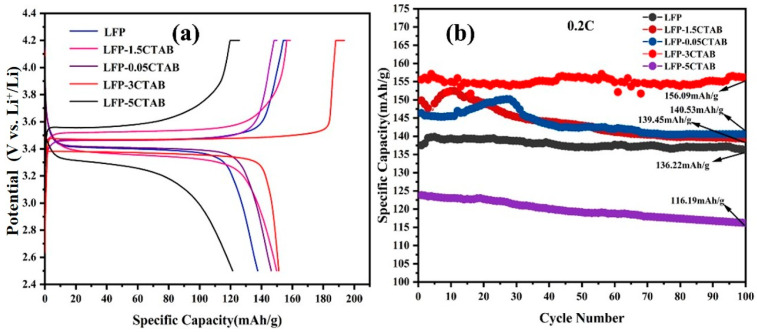
Charge/discharge and cycling graphs of lithium iron phosphate modified with different amounts of CTAB added to the precursor. (**a**) Initial charge/discharge curve. (**b**) Performance curve for 100 cycles.

According to Figure 11, the XRD pattern of the LiFePO_4_/C material obtained by further preparation of the FePO_4_ material obtained from the above experiments was compared with the standard PDF card (PDF #40-1499) progression, and no obvious stray peaks appeared. The SEM test of the resulting LiFePO_4_/C material is shown in Figure S9, which shows that the prepared LiFePO_4_/C material is consistent with the existing studies [20,21,22,23,24]. As can be seen from Figure S9, the lithium iron phosphate material consists of lamellar-stacked particles. Its maximum lamellar particle size is 400 nm, the minimum lamellar particle is 80 nm, and the average particle size is 280 nm. This makes it a relatively ideal lithium iron phosphate precursor. The carbon content in LiFePO_4_/C is exclusively derived from ascorbic acid pyrolysis as confirmed by the TGA mass loss (Figure 7). CTAB’s contribution is limited to precursor particle size reduction (250 nm for FP-3CTAB vs. 1.3 μm for unmodified FP, Figure 9), which enhances carbon coating uniformity and thereby improves cycling stability (99.36% capacity retention vs. 98% in controls, Figure 12b).

According to Figure 12, a comparative analysis was conducted on the initial charge/discharge performance of the lithium iron phosphate (LFP) prepared from distinct precursors [30,31,32]. The specific capacity of the LFP sample prepared from the FP sample without precursor modification was found to be 139 mA h g^−1^, the discharge plateau was found to be 3.39 V, and the specific capacity of the LFP sample after 100 charge/discharge cycles was 136 mA h g^−1^, with a capacity retention rate of 98%. The LFP-0.05CTAB sample prepared with the FP-0.05CTAB precursor exhibited a specific capacity of 143 mA h g^−1^ and a discharge plateau of 3.4 V at 0.2 C. After 100 charge/discharge cycles, the LFP sample demonstrated a capacity retention rate of 98%. The specific capacity of the LFP sample was 140 mA h g^−1^, with a capacity retention rate of 98.2%, which is an increase in the specific capacity of the sample compared to that of the LFP. The discharge point platform of the LFP-1.5CTAB prepared with FP-1.5CTAB was slightly decreased compared to LFP, which might be due to the lower amount of Fe^2+^ involved in the redox reaction, with the lower Fe-to-phosphorus ratio (0.914) of its precursor, which in turn led to the poorer electrochemical performance. The specific capacity of the LFP-3CTAB sample obtained from the FP-3CTAB precursor is 157 mA h g^−1^, and the discharge platform is 3.38 V at a 0.2C current. The specific capacity is 156 mA h g^−1^ after 100 charge/discharge cycles, with a capacity retention rate of 99.36%, which represents a significant improvement in the performance of LFP when compared with that of LFP, a known unmodified FP precursor. This enhancement can be attributed to the average particle size of FP-3CTAB, which is 250 nm. The lithium-ion diffusion channel of the LFP prepared from this precursor is shorter than that of the unmodified LFP, thereby improving its electrochemical performance. The specific capacity of the LFP prepared with FP-5CTAB as a precursor was 125 mA h g^−1^ at 0.2C, and the discharge platform was 3.23 V. After 100 charge/discharge cycles, the specific capacity of the LFP was 116 mA h g^−1^, and the capacity retention rate was 92.95%. A comparison with the unmodified LFP revealed that all performance metrics were reduced. This phenomenon can be attributed to the iron-to-phosphorus ratio in the FP-5 sample, which is only 0.8145. This results in a lower amount of Fe^2+^ participating in the redox reaction in the fabricated LFP, which in turn results in the poorer charge/discharge performance of the prepared LFP cathode material.

Electrochemical impedance spectroscopy (EIS) tests were performed on both pre-cycling and post-cycling LFP cells, with the results shown in Figure S12a. As shown in Figure S12a, an intercept at the Z’ axis at a high frequency corresponds to the ohmic resistance (R_s_), representing the resistance of the electrolyte (see the equivalent circuit model in Figure S13). The semicircle in the middle frequency range indicates the charge-transfer resistance (R_ct_). The inclined line in the low frequency region represents the Warburg impedance (W1), which relates to the solid-state diffusion of lithium ion inside the active particles. A constant phase element (CPE) was placed to represent the double layer capacitance and passivation film capacitance. The diffusion rate of Li+ in electrolyte solution is far greater than that of Li+ in solid-state active material, so the resistance of the charge transfer can be considered as the rate-determining step of the diffusion process of Li^+^ during the charge/discharge of a battery. Additionally, the cyclic voltammetry (CV) test results for the LFP cells are presented in Figure S12b. Post-cycling, a notable reduction in charge transfer resistance was observed in the LFP cell, indicating improved charge transfer kinetics and suggesting an enhanced lithium-ion diffusion coefficient. The CV test confirmed that the LFP cell fabricated using the experimentally prepared LFP material exhibited behavior consistent with the existing literature [8,20,21,22,23].

The solid-phase diffusion coefficient of Li^+^ is considered of significant interest due to its importance in improving the power density of lithium-ion batteries [33]. The determination of diffusion characteristics is dependent on the solution of the Warburg impedance response, and the diffusion coefficient ($D_{Li}^{+}$) of lithium ion can be calculated from the plots in the low frequency region according to the following equations:

(7) $$Z_{re}=R_{ct}+R_{s}+\sigma \omega^{-\frac{1}{2}}$$

(8) DLi+=R2T22A2n4F4CLi2σ2

where T is the absolute temperature, R the gas constant, n is the number of electrons per molecule during oxidization, A is the surface area, F is the Faraday’s constant, C_Li_ is the concentration of lithium ions, ω is the angular frequency, and σ is the Warburg factor which has a relationship with Z_re_. The Z_re_−ω^−1/2^ plots are presented in Figure S14. According to the results shown in Figures S12a and S14, the R_S_ post-cycling is 10.8 Ω; the R_ct_ post-cycling is 48 Ω. The lithium-ion diffusion coefficient ($D_{Li}^{+}$) was calculated from the Warburg impedance slope (Figure S14) using Equation (8): $D_{Li}^{+}$ = 1.2 × 10^−12^ cm^2^ s^−1^. This value aligns with the high-performance LFP cathodes reported in [33], confirming efficient Li^+^ transport enabled by the optimized precursor morphology. Moreover, these findings affirm that the presence of Ni in the experimental raw materials did not adversely affect the electrochemical performance of the LFP cells.

## 4. Conclusions

This study establishes a closed-loop materials strategy. Iron from low-grade laterite nickel ores is directly converted into FePO_4_ precursors via selective anodic oxidation; the optimized LiFePO_4_/C cathodes meet commercial performance benchmarks. In this study, we successfully optimized the synthesis of FePO_4_·2H_2_O via anodic oxidation by systematically adjusting reaction parameters and incorporating a CTAB surfactant. The optimized precursor exhibited controlled morphology, reduced particle size, the appropriate Fe/P ratio, and low impurity content. When used to synthesize LiFePO_4_/C cathodes, the optimized precursor led to a significantly improved electrochemical performance, demonstrating a higher specific capacity and excellent cycling stability. The specific capacity of the LFP-3CTAB sample obtained from the FP-3CTAB precursor is 157 mA h g^−1^, and the discharge platform is 3.38 V at a 0.2C current. The specific capacity is 156 mA h g^−1^ after 100 charge/discharge cycles, with a capacity retention rate of 99.36%, which represents a significant improvement in the performance of LFP when compared with that of LFP, a known unmodified FP precursor.

## Figures and Tables

**Table 1:** The main chemical components (wt.%) of nickel–iron alloy.

Chemical Element	Fe	Ni	Mn	Si	C
Fe_0.64_Ni_0.36_	64.01	35.98	0.59	0.31	0.05

**Table 2:** Uniform design experiment factor level table.

Factor	Electrolyte Concentration (mol/L)	Voltage (V)	pH	Electrolysis Time (h)
1	4	8	6	3
2	7	4	1	9
3	3	2	5	2
4	5	1	9	4
5	8	3	4	7
6	1	6	2	5
7	2	5	7	1
8	9	7	8	6
9	6	9	3	8

**Table 3:** Uniform experimental design scheme.

Scheme	Electrolyte Concentration (mol/L)	Voltage (V)	pH	Electrolysis Time (h)	Temperature (°C)
FP-1	4(0.7)	8(15)	6(2.5)	3(3)	60 °C
FP-2	7(1.5)	4(11)	1(0.8)	9(9)	60 °C
FP-3	3(0.5)	2(9)	5(2.2)	2(2)	60 °C
FP-4	5(1.0)	1(8)	9(3.2)	4(4)	60 °C
FP-5	8(1.7)	3(10)	4(2.2)	7(7)	60 °C
FP-6	1(0.1)	6(13)	2(1.3)	5(5)	60 °C
FP-7	2(0.3)	5(12)	7(2.6)	1(1)	60 °C
FP-8	9(2.0)	7(14)	8(3.0)	6(6)	60 °C
FP-9	6(1.2)	9(16)	3(1.6)	8(8)	60 °C

**Table 4:** Single-factor experimental design.

Electrolyte Concentration (mol/L)	Voltage (V)	pH	Electrolysis Time (h)	Water Bath Temperature (°C)	CTAB Dosage (%)
1.2	16	1.6	8	60	0.05
1.2	16	1.6	8	60	1.5
1.2	16	1.6	8	60	3
1.2	16	1.6	8	60	5

## Data Availability

The original contributions presented in this study are included in the article and Supplementary Materials. Further inquiries can be directed to the corresponding authors.

## Supplementary Materials

The following supporting information can be downloaded at: https://www.mdpi.com/article/10.3390/ma18112555/s1.

## Abbreviations

The following abbreviations are used in this manuscript:

LFP	LiFePO_4_
CTAB	Cetyltrimethylammonium bromide
FP	FePO_4_
EIS	Electrochemical impedance spectroscopy
CV	Cyclic Voltammetry
pH	Degree of acidity or alkalinity
LFP/C	LiFePO_4_/C
SCE	Saturated Calomel Electrode
LIBs	Lithium-ion batteries
