# Supplementary material for: Process Optimization for the Preparation of the Lithium Iron Phosphate Precursor FePO4·2H2O by Anodic Oxidation Method
Source: Materials (Basel). 2025 May 29;18(11):2555. doi: 10.3390/ma18112555 (PMC12155975; doi:10.3390/ma18112555)
Supplement: Supplementary file 1 [file materials-18-02555-s001.zip › materials-3558982-supplementary.pdf]

materials

MDPI

*Supplemental Files*

# Process Optimization for the Preparation of Lithium Iron Phosphate Precursor $FePO_4 \cdot 2H_2O$ by Anodic Oxidation Method (Supplemental Files)

**Table S1 Elemental Composition at Various Temperature Conditions**

| Temperature | Elemental | Quantity contained (mg/kg) | Fe/P | $Ni^{2+}$ quantity contained (%) |
|---|---|---|---|---|
| | Fe | 395400 | | |
| 40°C | P | 206700 | 1.05 | 0.1303 |
| | Ni | 1303 | | |
| | Fe | 396500 | | |
| 50°C | P | 204700 | 1.07 | 0.2490 |
| | Ni | 2489 | | |
| | Fe | 311000 | | |
| 60°C | P | 172000 | 1.00 | 0.1268 |
| | Ni | 1268 | | |
| | Fe | 287600 | | |
| 70°C | P | 176200 | 0.902 | 0.1769 |
| | Ni | 1769 | | |

Table S2 Comparison Table of Element Content of Samples FP1~FP-9 Obtained from Uniform Experimental Design

| Sample Name | Elemental | Quantity contained (mg/kg) | Fe/P | $Ni^{2+}$quantity contained (%) |
|---|---|---|---|---|
| | Fe | 251400 | | |
| FP-1 | P | 161300 | 0.865 | 0.24 |
| | Ni | 2478 | | |
| | Fe | 221300 | | |
| FP-3 | P | 161200 | 0.75 | 0.0896 |
| | Ni | 895 | | |
| | Fe | 283400 | | |
| FP-4 | P | 176500 | 0.88 | 0.2217 |
| | Ni | 2217 | | |
| | Fe | 403000 | | |
| FP-5 | P | 236800 | 0.944 | 0.31 |
| | Ni | 3232 | | |
| | Fe | 260400 | | |
| FP-6 | P | 185400 | 0.76 | 0.1304 |
| | Ni | 1304 | | |
| | Fe | 287600 | | |
| FP-8 | P | 176200 | 0.903 | 0.05 |
| | Ni | 450 | | |

| | | | | |
|---|---|---|---|---|
| **FP-9** | **Fe** | **311800** | **1.006** | **0.14** |
| | **P** | **171200** | | |
| | **Ni** | **1440** | | |

ICP-OES was used to analyze and test the content of elements in FP1~9 and impurity Ni in the samples obtained from this homogenization experimental design and to calculate the iron and phosphorus ratio in each sample, and the content of each element in the samples obtained as well as the iron and phosphorus ratios are shown in Table S1.

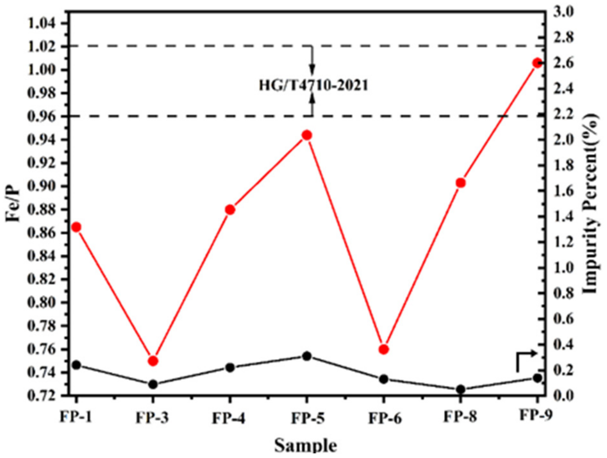


Figure S1 Iron phosphorus ratio and impurity content of the sample obtained from homogenization experimental design.

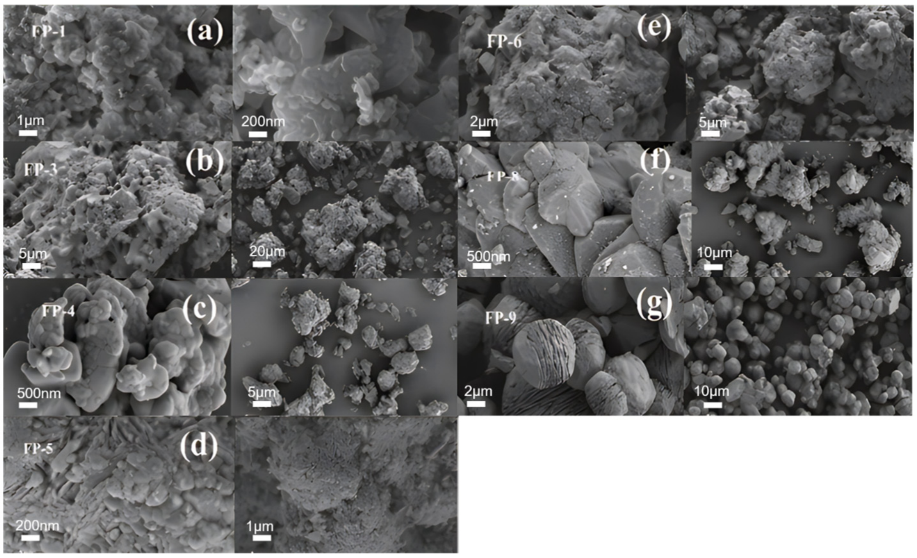


**Figure S2 SEM images of the products of the homogenization experiment: (a) FP-1, (b) FP-2, (c) FP-4, (d) FP-5, (e) FP-6, (f) FP-8, and (g) FP-9.**

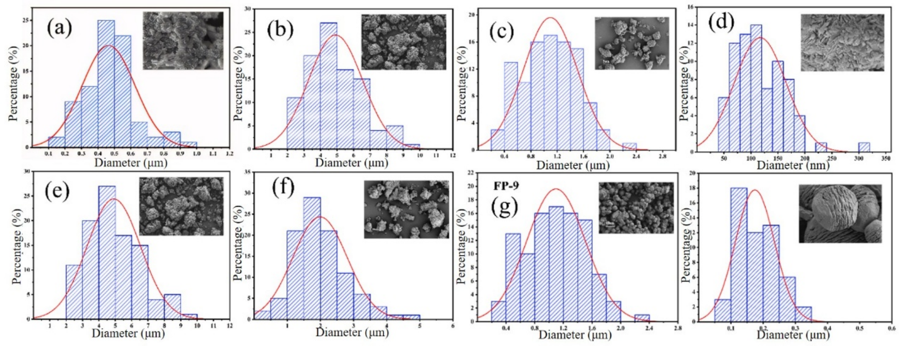


**Figure S3 Particle size distributions of the anodic oxidation products from the homogenization experiment: (a) FP-1, (b) FP-3, (c) FP-4, (d) FP-5, (e) FP-6, (f) FP-8, and (g) FP-9.**

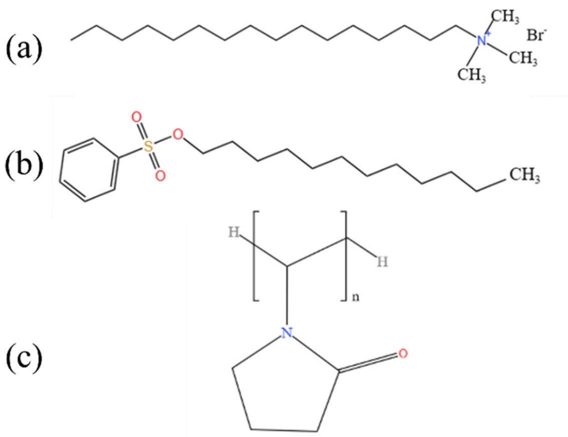


**Figure S4 Structures of surfactants (a) CTAB (b) SDBS (c) PVP**

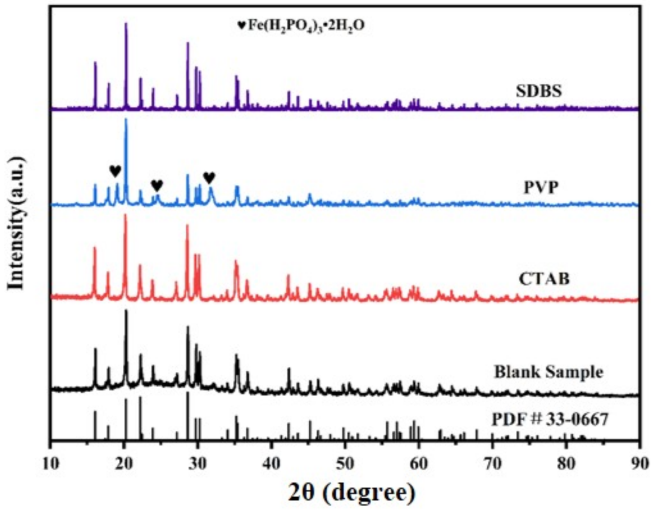


**Figure S5 XRD patterns of iron phosphate prepared with different surfactants**

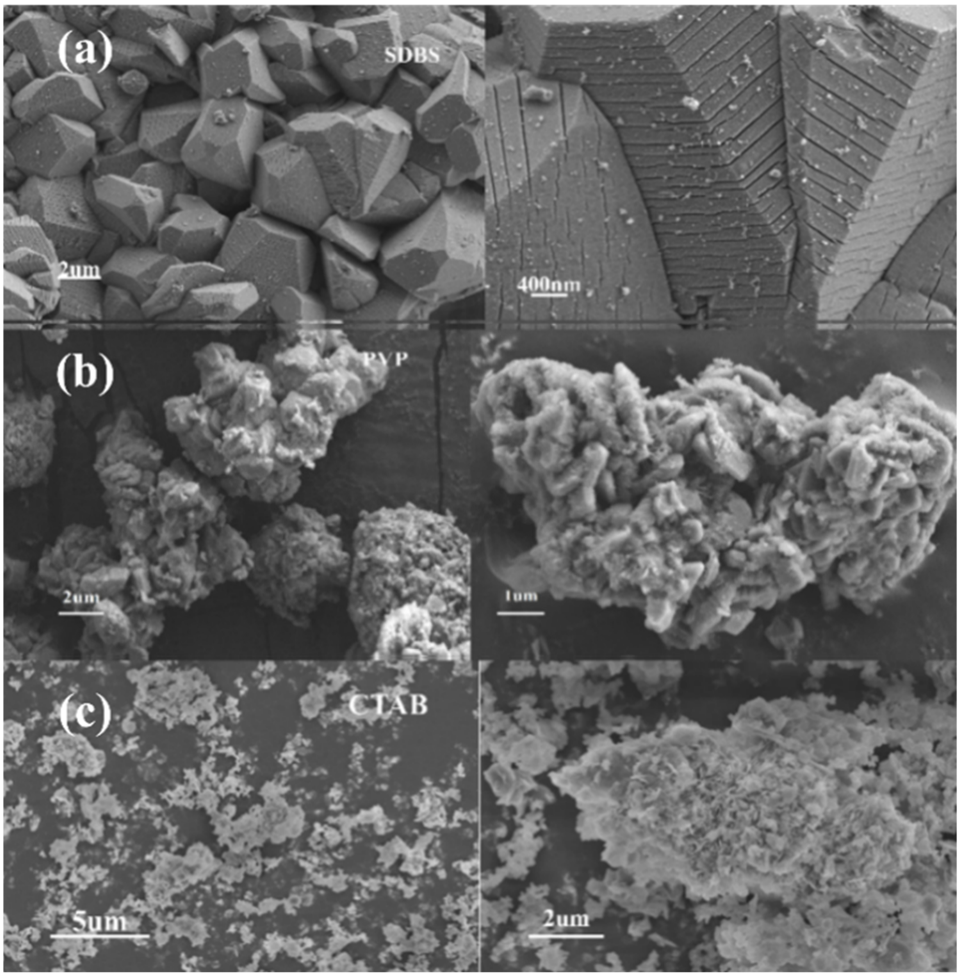


**Figure S6 SEM images of anodic oxidation products obtained with the addition of different surfactants: (a) SDBS-$FePO_4$ (b) PVP-$FePO_4$ (c) CTAB-$FePO_4$.**

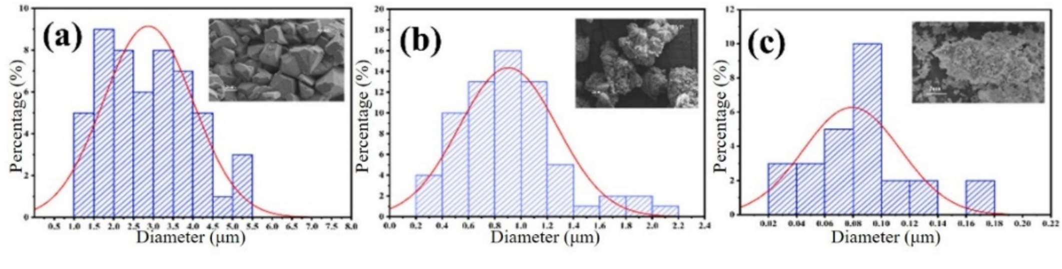


**Figure S7 Particle size distribution of iron phosphate under the condition of adding different surfactants (a) SDBS-$FePO_4$ (b) PVP-$FePO_4$ (c) CTAB-$FePO_4$**

Table S3 Comparison of element contents in samples from FP-0.05CTAB to FP-5CTAB obtained through single-factor experimental design

| Sample Name | Elemental | Quantity contained (mg/kg) | Fe/P | $Ni^{2+}$quantity contained (%) |
|---|---|---|---|---|
| | Fe | 310800 | | |
| FP-0.05CTAB | P | 170200 | 1.009 | 0.154 |
| | Ni | 1540 | | |
| | Fe | 296500 | | |
| FP-1.5CTAB | P | 180300 | 0.914 | 0.11 |
| | Ni | 1081 | | |
| | Fe | 254700 | | |
| FP-3CTAB | P | 141400 | 1.001 | 0.0769 |
| | Ni | 769 | | |
| | Fe | 241800 | | |
| FP-5CTAB | P | 164200 | 0.815 | 0.3192 |
| | Ni | 3192 | | |

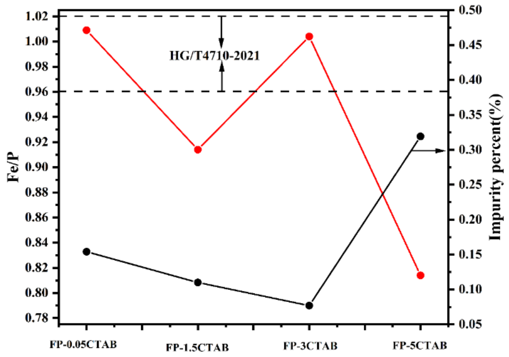


**Figure S8 Iron to Phosphorus Ratio and Impurity Content in Samples Obtained from Single-Factor Experimental Design.**

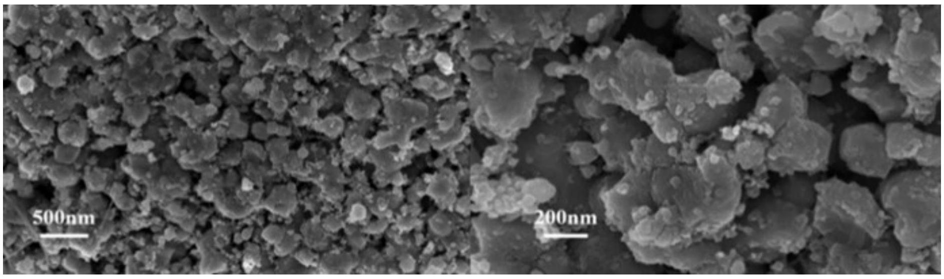


**Figure S9 SEM images of lithium iron phosphate.**

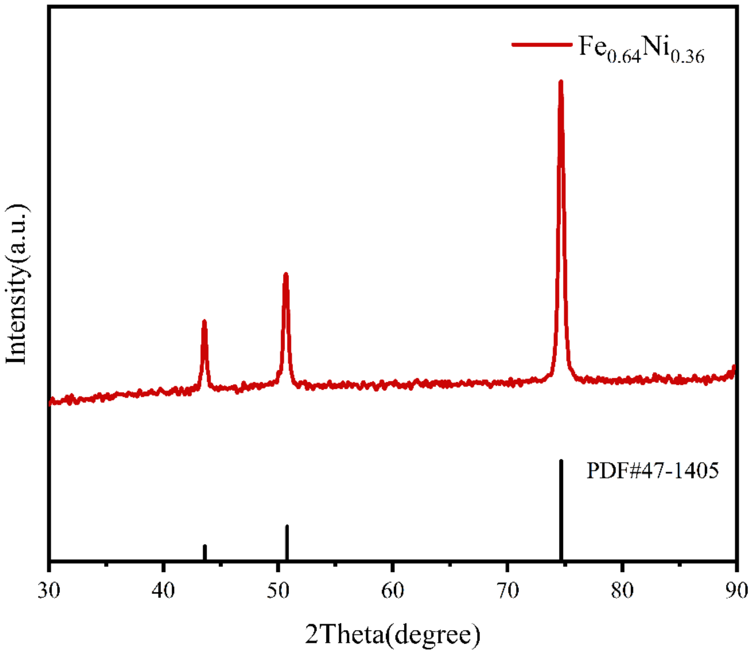


**Figure S10 XRD pattern of Nickel-Iron Alloy.**

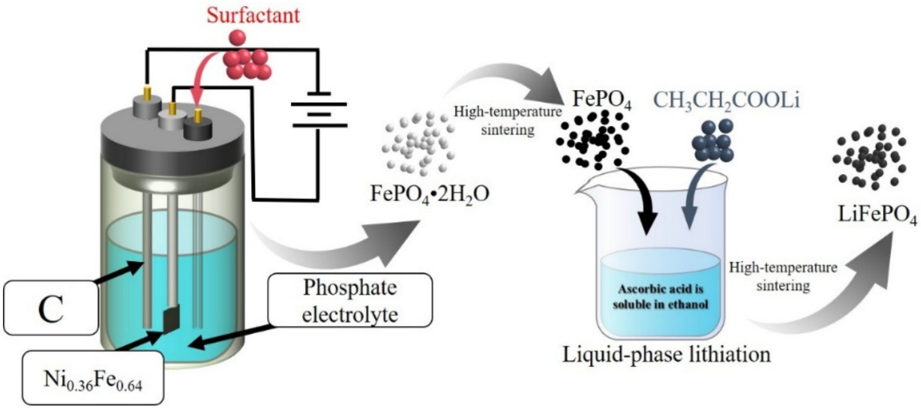


**Figure S11 Flowchart of the experiment.**

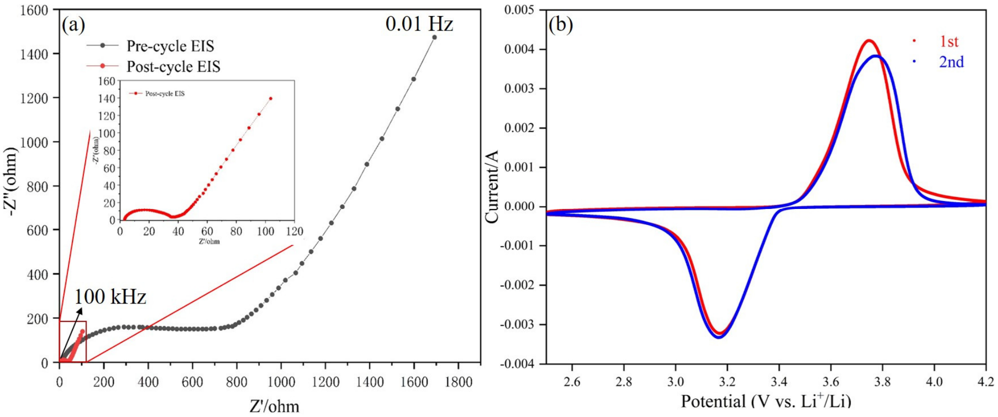


**Figure S12. CV and EIS tests of LFP cells obtained from the optimal experimental conditions scheme (a) Pre-cycling and Post-cycling EIS tests (b) CV curves at a scan rate of 0.5 mV s$^{-1}$ for the first two cycles in a 2.5–4.2V voltage window.**

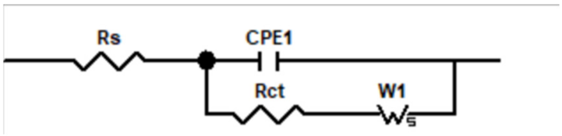


**Figure S13. Equivalent circuit to fit with the impedance data**

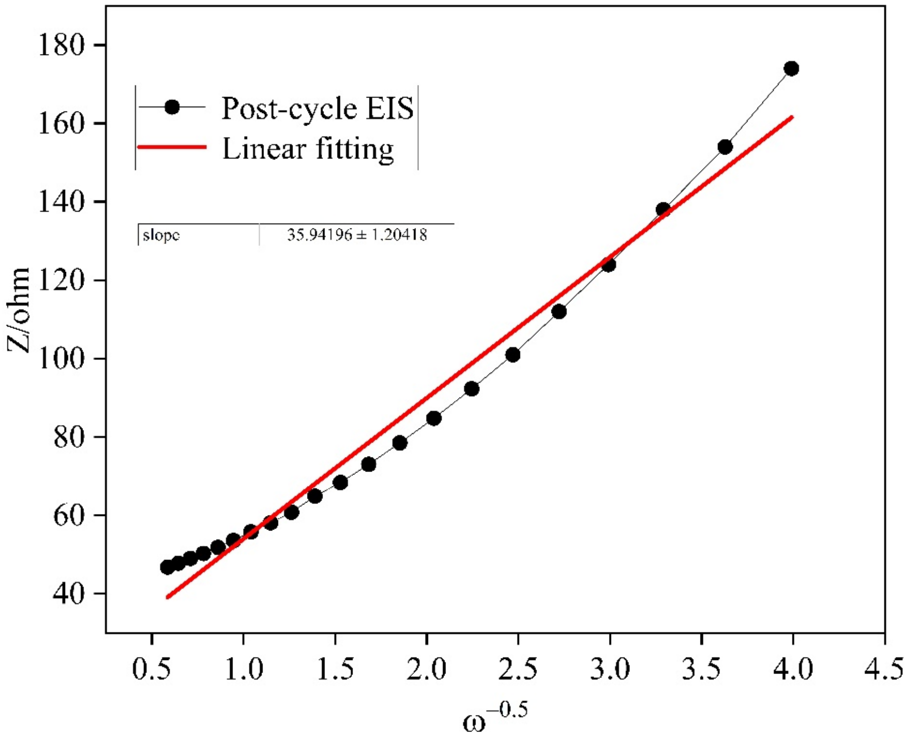


**Figure S14. The fitting curves of $Z_{re}$ and $\omega^{-1/2}$ of Post-cycling EIS**
